# d-ribose induces nephropathy through RAGE-dependent NF-κB inflammation

**DOI:** 10.1007/s12272-018-1061-z

**Published:** 2018-08-13

**Authors:** Jinni Hong, Xuemei Wang, Ning Zhang, Hong Fu, Weiwei Li

**Affiliations:** 1 Integrated Laboratory of Traditional Chinese Medicine and Western Medicine, Peking University First Hospital, Beijing, 100034 People’s Republic of China; 2 Department of Pharmacology and Toxicology, School of Medicine, Virginia Commonwealth University, Richmond, VA 23298 USA

**Keywords:** Diabetes, AGEs, RAGE, Kidney

## Abstract

Recently, aberrantly high levels of d-ribose have been discovered in type II diabetic patients. d-ribose glycates proteins more rapidly than d-glucose, resulting in the production of advanced glycation end products (AGEs). Accumulations of these products can be found in impaired renal function, but the mechanisms are poorly understood. The present study tested whether d-ribose induces renal dysfunction via the RAGE-dependent NF-κB signaling pathway. In vivo, administration of d-ribose was found to lower blood glucose and regulate insulin tolerance. Compared to controls, urine nitrogen and creatinine excretion were increased in mice receiving d-ribose and were accompanied by severe pathological renal damage. Furthermore, immunohistochemistry showed that NF-κB, AGEs, and receptor of AGEs (RAGE) increased in the kidneys of the mice with d-ribose treatment. In vitro, by western blot and immunofluorescent staining, we confirmed that d-ribose induced NF-κB activation and accumulation of AGEs and RAGE in mesangial cells. By co-immunoprecipitation, we found that the pull-down of RAGE remarkably increased the expression of NF-κB. Silencing the RAGE gene blocked the phosphorylation of NF-κB induced by d-ribose. These results strongly suggest that d-ribose induced NF-κB inflammation in a RAGE-dependent manner, which may be a triggering mechanism leading to nephropathy.

## Introduction

In 1970, d-ribose was found to be an energy enhancer that decreases blood glucose (Steinberg et al. 1970). In its role as a sugar moiety of adenosine triphosphate (ATP), d-ribose is widely used as a metabolic therapy supplement for chronic fatigue syndrome or cardiac energy metabolism (Segal et al. 1957). However, d-ribose is more active than glucose in protein glycation (Lu and He 2014; Wei et al. 2012; Chen et al. 2010). It glycates proteins and produces advanced glycation end products (AGEs) that have severe cytotoxicity, which can lead to cell dysfunction and death. Administration of d-ribose leads to high yields of AGEs in the mouse brain and subsequently impairs mouse spatial cognition (Han et al. 2011). Evidence has demonstrated a potential role of AGEs in the progression of renal dysfunction (Chilelli et al. 2013). Cellular proteolysis of AGEs-modified proteins forms AGEs-free adducts, which accumulate markedly in plasma with a decline in the glomerular filtration rate (Agalou et al. 2005). It has been proposed that AGEs bind specifically to cell surface receptors to activate cell dysfunction (Fehrenbach et al. 1998). The receptor for advanced glycation end products (RAGE) is one of the receptors closely correlated with nephropathy (Fehrenbach et al. 1998; Hou et al. 2004; Tanji et al. 2000). Mice that overexpressed RAGE showed severe renal dysfunction compared to their littermates or to RAGE-gene knockout mice (Sourris et al. 2010). These findings suggested that the blockage of AGEs and RAGE accumulations improves renal dysfunction (Wendt et al. 2003). However, whether d-ribose induces AGEs and RAGE accumulation in the kidney with consequential renal dysfunction is still unknown.

Accumulating evidence about the pathogenesis of nephropathy indicates that activation of the nuclear factor-kappa B (NF-κB) signaling pathway is involved in the progression of renal dysfunction, including minimal change disease and diabetic nephropathy (Mezzano et al. 2001, 2004). NF-κB helps to control the expression of numerous genes encoding proteins involved in immune and inflammatory responses. Specifically, it is activated in nephropathy (Navarro and Mora 2005; Costa et al. 2006), inhibiting it attenuates the activation of interstitial fibroblasts (Tamada et al. 2003), and it is induced by various cellular stress-associated stimuli such as reactive oxygen species (ROS) (Karin and Greten 2005). d-ribose reacts with β2-microglobulin and induced ribosylated protein via a ROS-mediated pathway (Kong et al. 2011). However, to our knowledge, until now no study has linked the detrimental effect of d-ribose to the activation of NF-κB inflammation in the development of nephropathy.

The present study was designed to test the hypothesis that d-ribose induces NF-κB inflammation in a RAGE-dependent manner, which may be a triggering mechanism leading to nephropathy. We first tested whether d-ribose would induce renal damage in mice. Next, we tested whether the mechanism of nephropathy induced by d-ribose is associated with the AGEs, RAGE, and NF-κB signaling pathways. Then, we performed in vitro experiments to test whether RAGE is required for the NF-κB activation induced by d-ribose.

## Materials and methods

### Animal grouping and treatment

Male BABL/c mice (n = 45, 8–10 weeks, 20 ± 2 g) were purchased from Vital River Laboratory Animal Technology (Beijing, China) [License No. SCXK (Jing) 2016-0011]. After 7 days of acclimatization to the laboratory environment, the BABL/c mice were randomly divided into three groups (n = 15), control, 1.6 g/kg d-ribose and 3.2 g/kg d-ribose groups, which respectively received daily intraperitoneal (i.p.) injections with 0.9% saline, 1.6 g/kg, or 3.2 g/kg d-ribose for 30 days. All mice were maintained in animal facilities under SPF conditions. All animal experiments were performed with the approval of the Institutional Animal Care and Use Committee of Peking University First Hospital (Approval Number: J201534).

### Blood glucose and renal function analysis

Blood glucose was assessed using the glucose oxidase method; the OGTT (oral glucose tolerance test) and ITT (insulin tolerance test) were performed as described previously (Hong et al. 2017; Wu et al. 2018). Urea nitrogen (UN) and creatinine (Cr) in serum were measured with urease methods and sarcosine oxidase methods, respectively, both using commercial detecting kits according to the manufacturer’s protocol.

### Ultrastructural analysis, histopathological analysis, and immunohistochemistry analysis

After treatment for 30 days, the mice were sacrificed, and the kidneys were removed. Ultrastructural analysis, hematoxylin and eosin (HE), periodic acid-Schiff (PAS) staining, AGEs (Abcam, UK), RAGE (Abcam, UK) and NF-κB (Cell Signaling Technology, USA) staining were performed and quantified as described (Hong et al. 2017).

### Cell culture and treatment

The SV40 MES 13 cell line (mouse renal glomerular mesangial cells, MSCs) was purchased from the China Center for Type Culture Collection cell bank (China) and cultured in low-glucose DMEM (Gibco, Rockville, MD, USA) and F-12 medium (Gibco, Rockville, MD, USA) (low-glucose DMEM:F-12 medium = 3:1; final concentration, 6.7 mM glucose) supplemented with 5% FBS (Invitrogen), 100 U/ml penicillin, and 100 μg/ml streptomycin (Sigma-Aldrich, St. Louis, MO, USA). The cells were incubated with high glucose (high-glucose DMEM:F-12 medium = 3:1; final concentration, 25 mM glucose) or d-ribose (25 mM and 50 mM) for 48 h after an initial 24 h incubation in non-serum 6.7 mM glucose.

### Western blot

After the kidneys were collected from the mice, an equivalent of protein was resolved on dodecyl sulfate (SDS)-polyacrylamide gels and transferred to polyvinylidene difluoride (PVDF) membranes. The antibodies used are shown below: AGEs and RAGE antibodies were from Abcam, UK; p-IKKα/β, IKKβ, IKKα, p-IκBα, IκBα and NF-κB p65 antibodies were from Cell Signaling Technology, USA; p-NF-κB p65 antibody was from Santa Cruz Biotechnology, Glostrup, Denmark and GAPDH antibody was from Beijing Zhong Shan Golden Bridge Biotechnology Co., Ltd., China, with a dilution of 1:1000. Horseradish peroxidase-labeled secondary antibody was from Beijing Zhong Shan Golden Bridge Biotechnology Co., Ltd., with a dilution of 1:10,000. The membranes were developed with enhanced chemiluminescence (Thermo Scientific, USA) and visualized using a digital imaging system (BIO-RAD Laboratories, Inc., USA).

### ELISA

Sera were collected after treatment for 10, 20, and 30 days. Insulin (ALPCO, USA), AGEs (Abbexa Ltd., UK), and RAGE (Bio-Techne, USA) in serum were measured using commercial ELISA kits according to the manufacturer’s protocol.

### Immunofluorescence staining

After treatment, the cells were fixed, blocked, and stained with NF-κB antibody overnight at 4 °C. After incubation with a secondary antibody (1:400) conjugated to fluorescein isothiocyanate (FITC, Zhongshan Golden Bridge Biotechnology) for 1 h at room temperature, the cells were mounted with diamidinophenylindole (DAPI) and then observed and photographed with fluorescent microscopy (ECLIPSE TE2000-S, Nikon, Japan).

### Co-immunoprecipitation (Co-IP)

Co-IP was performed to confirm the interaction between RAGE and NF-κB. Cells were seeded in 10 cm dishes and grown to 80% confluence. After 24 h of non-serum starvation, the cells were incubated with or without 50 mM d-ribose for 48 h for co-IP. After washing three times with PBS, 1 mg cellular protein was lysed in 500 ul of cold cell lysis buffer and then incubated with RAGE antibody (Santa Cruz Biotechnology, Glostrup, Denmark) for 6 h. The immunocomplex was captured by adding 30 μl of protein G (Miltenyi Biotec, Germany) and subjected to western blot analysis (anti-NF-κB antibody).

### RNA interference of RAGE

RAGE small interference RNAs (siRAGE) were purchased from Santa Cruz Biotechnology, Glostrup, Denmark. SiRAGE was performed with silent Lipid Reagent (Bio-Rad, USA) according to the manufacturer’s instructions. The final concentration of siRAGE was 10 nM.

### Data analysis

All the data were analyzed using SPSS 19.0 statistical software and reported as the mean ± standard deviation (SD). Different groups were analyzed using a one-way analysis of variance (ANOVA) with Dunnett’s multiple comparison post-test. *P *< 0.05 was considered statistically significant.

## Results

### d-ribose down-regulated blood glucose and induced renal damage in mice

**Fig. 1:**
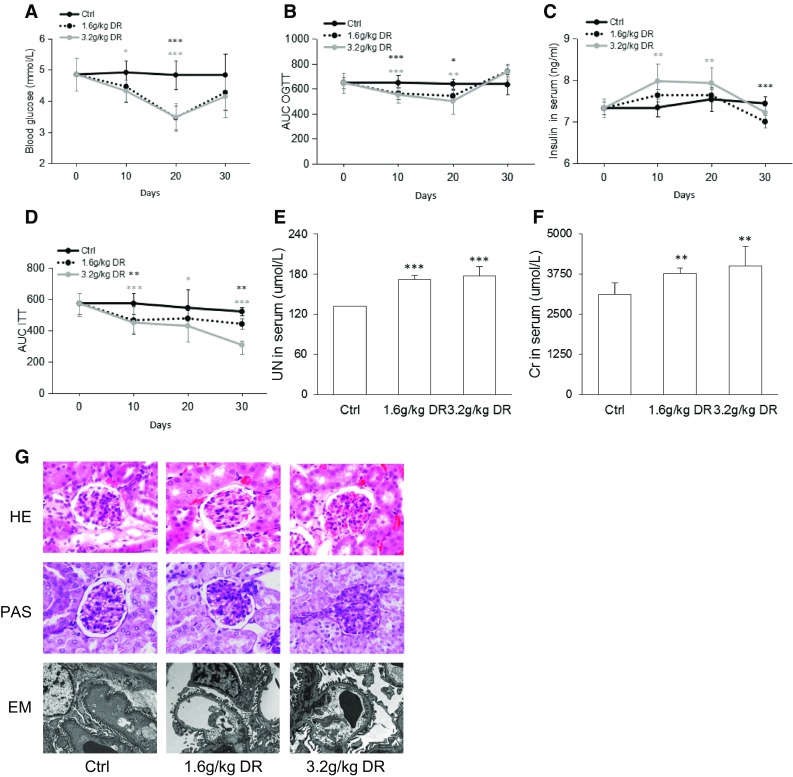
d-ribose down-regulated blood glucose and induced renal damage in mice. **a** Fasting blood glucose of mice (n = 5). **b** Area under the OGTT curve of mice (n = 5). **c** Insulin level in serum of mice (n = 5). **d** Area of ITT of mice (n = 5). **e** Urine nitrogen level in the serum of mice after 30 days of treatment (n = 5). **f** Creatinine level in the serum of mice after 30 days of treatment (n = 5). **g** Representative images of hematoxylin and eosin (HE) staining (original magnification ×400), periodic acid-Schiff (PAS) staining (original magnification ×400) and electron microscopy (EM) (original magnification ×400) analysis of mouse kidney after 30 days of treatment (n = 5). *Ctrl* control, *DR* d-ribose. Data are expressed as mean ± SD; *P < 0.05; **P < 0.01; ***P < 0.001 compared to the control group

We first investigated whether the d-ribose treatment affected glucose and insulin tolerance in the mice. BABL/c mice were i.p. injected with 1.6 g/kg or 3.2 g/kg d-ribose for 30 days. As shown in Fig. 1a, the fasting blood glucose in the d-ribose-treated mice significantly decreased after treatment for 10 and 20 days in comparison with the control group, confirming that d-ribose down-regulated blood glucose in mice (Segal et al. 1957). Furthermore, an OGTT analysis was performed to determine whether d-ribose affects glucose tolerance. After the d-ribose treatment for 10 or 20 days, the area under the curve of the OGTT was decreased but returned to the normal level after 30 days of treatment (Fig. 1b). These changes indicated that d-ribose ameliorated glucose tolerance in the shorter time periods (10 and 20 days). To further detect whether d-ribose played a role in insulin tolerance, the insulin level in serum and ITT analysis was measured. As shown in Fig. 1c, the insulin level in serum was up-regulated after treatment for 10 and 20 days and returned to a normal level after that. The area under the curve of the ITT decreased in a dose-dependent manner compared to the controls (Fig. 1d). All these suggested that d-ribose might be beneficial for insulin tolerance.

To investigate whether d-ribose induces renal dysfunction, UN and Cr in serum were measured. As shown in Fig. 1e and f, d-ribose induced severe levels of UN and Cr in serum in a dose-dependent manner. We further observed morphological changes microscopically (Fig. 1g). The HE staining showed that plasma proteins had been extravagated from the renal capsules and that the glomerular capillaries were dilated and filled with red blood cells, indicating more severe pathological changes in the kidneys of the mice who had undergone the d-ribose treatment than in the controls. The PAS staining showed a variety of typical glomerular damage, including severe basement membrane thickening, glomerular hypertrophy, and increased mesangial matrix area in the mice receiving d-ribose. Furthermore, electron microscopy of the renal cortex showed mesangial matrix deposition, mesangial expansion, podocyte fusion, and glomerular basement membrane thickening in the mice treated with d-ribose. All these changes were less severe in the control group, indicating that d-ribose accelerated renal dysfunction.

### d-ribose activated NF-κB signaling pathway in mice

**Fig. 2:**
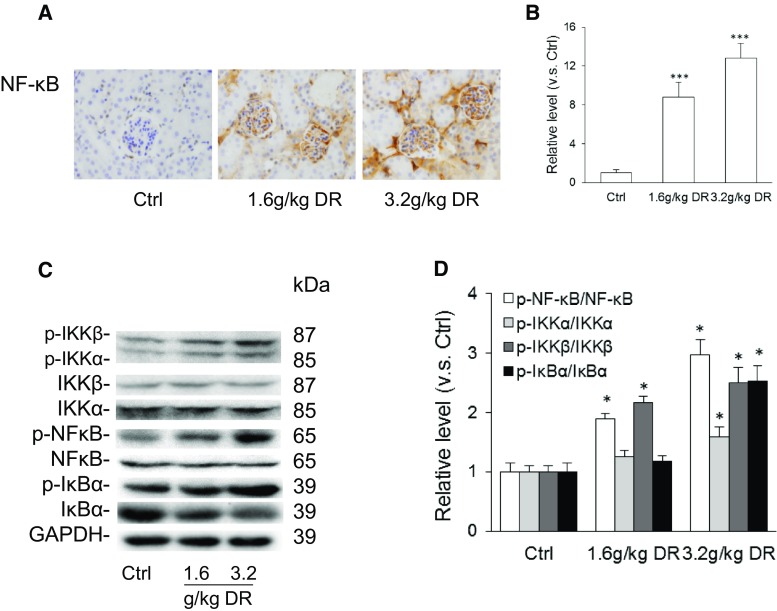
d-ribose activated NF-κB signaling pathway in mice. **a** and **b** Representative images (original magnification ×400) and summarized data of immunohistochemical stained kidney for NF-κB production in mice (n = 5). **c** and **d** Representative western blot gel document and summarized data from AGEs, RAGE, p-IKKβ, p-IKKα, IKKβ, IKKα, p-NF-κB, NF-κB, p-IκBα, and IκBα in kidney (n = 5). *Ctrl* control, *DR* d-ribose. Data are expressed as mean ± SD; *P < 0.05; **P < 0.01; ***P < 0.001 compared to the control group

To test whether d-ribose initiates a cellular inflammatory response through the NF-κB pathway, we evaluated the NF-κB signaling pathway activity in mice. The results of immunohistochemistry staining showed elevated NF-κB accumulation in the kidneys of the mice receiving d-ribose in a dose-dependent manner (Fig. 2a, b). As depicted in Fig. 2c and d, a Western blot showed up-regulated expression of phosphorylated IKKβ, IKKα, NF-κB p65, and IκBα with d-ribose treatment in comparison with the controls, indicating that the NF-κB signaling pathway was activated with d-ribose treatment.

### d-ribose enhanced AGEs and RAGE accumulation in mice

**Fig. 3:**
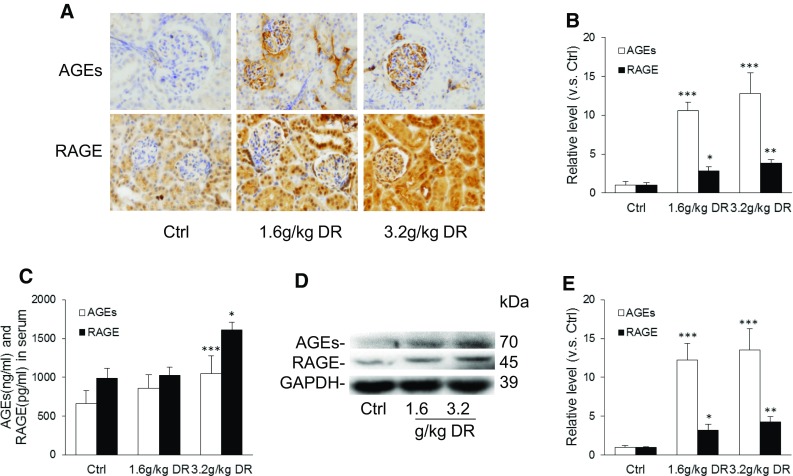
d-ribose enhanced AGEs and RAGE accumulation in mice. **a** and **b** Representative images (original magnification ×400) and summarized data of immunohistochemical stained kidney for AGEs and RAGE production in (n = 5). **c** AGEs and RAGE levels in the serum of mice after 30 days’ treatment (n = 5). **d** and **e** Representative western blot gel document and summarized data of AGEs and RAGE in kidney (n = 5). *Ctrl* control, *DR* d-ribose. Data are expressed as mean ± SD; *P < 0.05; **P < 0.01; ***P < 0.001 compared to the control group

Based on a previous study, AGEs and RAGE played a vital role in the progression and aggravation of chronic kidney disease (CKD) and end-stage renal disease (ESRD) (Gugliucci and Menini 2014). The present study examined the effect of d-ribose on AGEs and RAGE accumulation. By immunohistochemistry, AGEs and RAGE accumulations were found remarkably elevated in the kidneys of the mice with d-ribose treatment in a dose dependent manner in comparison with the controls (Fig. 3a, b). Consistently, AGEs and RAGE levels in serum (Fig. 3c) as well as AGEs and RAGE protein expression (Fig. 3d, e) in the kidneys were up-regulated in the mice receiving d-ribose treatment as compared to the controls. All these findings confirmed that d-ribose induced AGEs and RAGE accumulations (Han et al. 2011).

### d-ribose induced AGEs and RAGE enhancement and NF-κB activation in mesangial cells

**Fig. 4:**
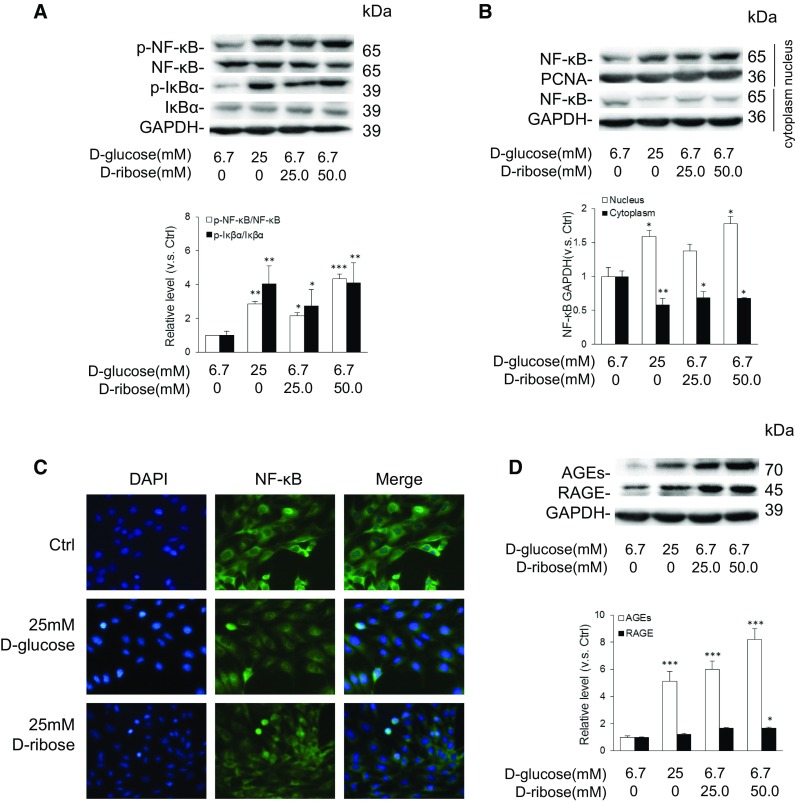
d-ribose induced AGEs and RAGE enhancement and NF-κB activation in mesangial cells. **a** Representative western blot gel document and summarized data of p-NF-κB, NF-κB, p-IκBα, and IκBα in mesangial cells (n = 5). **b** Representative western blot gel document and summarized data of NF-κB in nucleus and cytoplasm of mesangial cell (n = 5). **c** Representative images of immunostained mesangial cells for NF-κB (original magnification ×200) (n = 5). **d** Representative western blot gel document and summarized data of AGEs and RAGE in mesangial cells (n = 5). Data are expressed as mean ± SD; *P < 0.05; **P < 0.01; ***P < 0.001 compared to the control group

To further elucidate the effect of d-ribose on AGEs, RAGE, and NF-κB, we performed experiments in vitro with MSCs. The activity of NF-κB, phosphorylation of total NF-κB, and NF-κB in the nucleus and cytoplasm, were each detected, and immunofluorescence staining of NF-κB was performed. As depicted in Fig. 4a, both d-glucose and d-ribose up-regulated the expression of phosphorylated NF-κB and IκBα. NF-κB was expressed more prominently in the nucleus than the cytoplasm after d-glucose or d-ribose incubation in comparison with the controls (Fig. 4b). Consistently, co-localization of NF-κB and DAPI showed that in MSCs, NF-κB expressed more in the nucleus than the cytoplasm after incubation with d-glucose or d-ribose (Fig. 4c). All these results indicated that d-ribose activated NF-κB signaling pathways in MSCs.

As shown in Fig. 4d, the western blot gel document showed that both d-glucose and d-ribose up-regulated AGEs and RAGE expression. MSCs incubated with d-ribose showed a higher expression of AGEs and RAGE compared to d-glucose; this is consistent with studies that showed that d-ribose gives rise to AGEs more quickly than d-glucose (Wei et al. 2012; Han et al. 2011).

### RAGE played a vital role in d-ribose-induced NF-κB activation

**Fig. 5:**
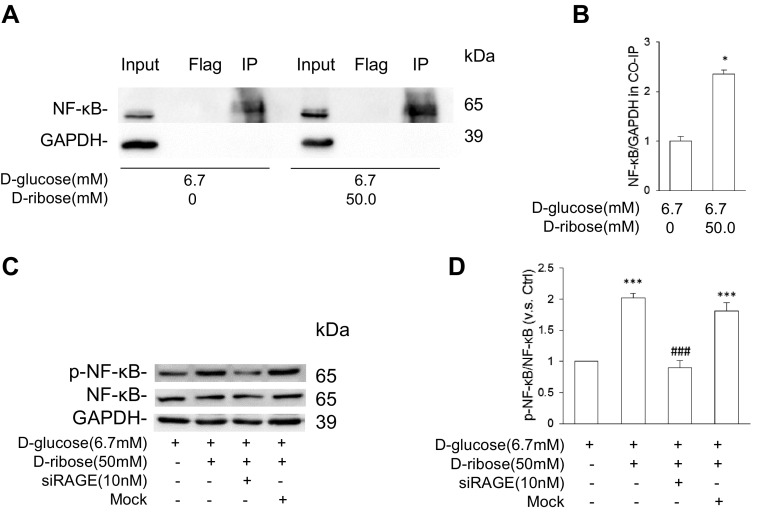
RAGE played a vital role in d-ribose-induced NF-κB activation. **a** and **b** Representative western blot gel document and summarized data of co-IP demonstrating a direct interaction of RAGE and NF-κB in mesangial cell (n = 5). **c** and **d** Representative western blot gel document and summarized data of p-NF-κB and NF-κB in mesangial cell with siRAGE pre-treatment (n = 4). Data are expressed as mean ± SD; *P < 0.05; **P < 0.01; ***P < 0.001 compared to the control group

To determine whether RAGE is correlated to NF-κB activation, co-IP with RAGE antibody was performed, and the NF-κB expression was evaluated. MSCs were treated with or without 50 mM d-ribose for 48 h and then co-IPed with RAGE antibody. As shown in Fig. 5a and b, the expression of NF-κB was higher in MSC treated with d-ribose than in the controls, indicating that d-ribose up-regulated the expression of NF-κB and that RAGE is required in the process. To further confirm the role of RAGE in d-ribose-induced NF-κB activation, a siRNA against RAGE was transfected to MSCs prior to d-ribose incubation. As shown in Fig. 5c and d, siRAGE effectively down-regulated the expression of phosphorylated NF-κB induced by d-ribose according to the western blot analysis. All these results confirmed that d-ribose activated NF-κB in a RAGE-dependent way.

## Discussion

The present study was designed to determine whether d-ribose induced nephropathy through NF-κB-mediated inflammation and whether RAGE played a role in this NF-κB activation. In vivo, d-ribose was found to induce renal dysfunction and morphological changes in mice, with NF-κB activation, AGEs and RAGE accumulation in the kidneys. Furthermore, in vitro studies with mesangial cells showed that d-ribose indeed elevated AGEs and RAGE accumulation and NF-κB activation. The pull-down of RAGE remarkably up-regulated the expression of NF-κB, and the silencing of RAGE attenuated the accumulation of NF-κB induced by d-ribose. These findings suggested that RAGE-dependent NF-κB inflammation may play a vital role in nephropathy induced by d-ribose.

Previous studies have demonstrated that d-ribose elevated tau hyper-phosphorylation and Aβ-like deposits and caused memory loss and anxiety-like behavior in mice (Wu et al. 2015). However, whether d-ribose induces renal damage remains poorly studied. In the present study, we first demonstrated that d-ribose injections in mice induced severe urine nitrogen and creatinine excretion compared to the controls, accompanied by a series of characteristic pathological changes in the kidneys. To our knowledge, these results provided the first experimental evidence that d-ribose may induce renal damage.

It has been reported that inflammation plays a crucial role in a variety of inflammatory diseases including diabetic nephropathy (DN) (Park et al. 2011). As one of the important cellular mediators (Patel and Santani 2009; Kolati et al. 2015), NF-κB was found to be up-regulated in the glomeruli and tubules (Vallon 2011; Imig and Ryan 2013), suggesting a close correlation between NF-κB and nephropathy. Normally, NF-κB is inactive and present in cytoplasm, but once it is phosphorylated, free and active p-NF-κB migrates to the nucleus and regulates pro-inflammatory cytokines (DiDonato et al. 1997; Mercurio et al. 1997). NF-κB was observed to be transferred to the nucleus from the cytoplasm in mesangial cells treated with d-ribose using both immunofluorescent analysis and western blot analysis, indicating NF-κB signaling pathway activation. Phosphorylated-NF-κB serves as an important parameter of nucleus translocation. We also found an increased level of active p65-NF-κB in mesangial cells and in the mice treated with d-ribose. These results are consistent with previous reports that ribosylation-induced by d-ribose activates inflammation and astrocyte activation in the mouse brain (Han et al. 2014). Therefore, we hypothesized that NF-κB plays a crucial role in d-ribose-induced mesangial cell damage and renal damage.

d-ribose was first found to down-regulate blood glucose (Segal et al. 1957) and up-regulate insulin levels (Sloviter and Petkovic 1969), and thus “oral administration of d-ribose in diabetes mellitus” was described in 1957. However, when d-ribose is glycated, it gives rise to the formation of advanced glycation end products (AGEs) and RAGE in astrocytoma cells, resulting in direct damage to the nervous system (Patel and Santani 2009). However, there are almost no studies about the involvement of AGEs and RAGE in d-ribose-induced renal damage. The present study confirmed there is lower fasting blood glucose and higher insulin levels in the serum of mice treated with d-ribose. We also found significantly higher levels of AGEs and RAGE accumulations in the kidneys and mesangial cells treated with d-ribose in comparison with controls. d-glucose produced less AGEs and RAGE compared to d-ribose, confirming that d-ribose is a more reducing monosaccharide than d-glucose (Wei et al. 2009; Chen et al. 2009). The binding of RAGE by AGEs evokes a vicious cycle of increased oxidative stress and inflammatory reaction, leading to subsequent cell and tissue injury (Vlassara 2001), such as has been found in chronic renal diseases (Tanji et al. 2000). This led us to hypothesize that d-ribose activates NF-κB and induces mesangial cell damage and renal dysfunction, in which AGEs and RAGE play a role.

To test this hypothesis, a co-immunoprecipitation assay with RAGE and silencing RNA of RAGE were performed to test whether RAGE plays a role in the activation of NF-κB induced by d-ribose. Elevated NF-κB expression was found after a pull-down with RAGE, and NF-κB expressed more in MSCs treated with d-ribose in comparison with the controls, indicating that RAGE plays a role in NF-κB activation. Furthermore, prior treatment with siRAGE was confirmed to block the phosphorylation of NF-κB. In previous studies, the AGEs-RAGE pathway is widely recognized as a pro-inflammatory mechanism in nephropathy, and the therapeutic blockade of RAGE ameliorated renal and endothelial functions under a high AGEs burden (Yeh et al. 2017). Based on these results, it appears that RAGE is essential for the activation of NF-κB induced by d-ribose. However, in vivo experiments are needed to further confirm the role of RAGE in d-ribose-induced NF-κB activation in nephropathy.

In summary, the present study demonstrated d-ribose-induced mesangial cell damage and renal dysfunction in mice, a process which was mediated by the RAGE-dependent NF-κB signaling pathway. These findings imply a new mechanism for mediated d-ribose-induced nephropathy, providing glycation of d-ribose as a new target for the treatment of renal damage, especially nephropathy.
